# Infectious Knockdown of CREB and HIF-1 for the Treatment of Metastatic Uveal Melanoma

**DOI:** 10.3390/cancers11081056

**Published:** 2019-07-26

**Authors:** Hanna Voropaev, Maria Gimmelshein Vatkin, Dudi Shneor, Shahar Luski, Alik Honigman, Shahar Frenkel

**Affiliations:** 1 Division of Ophthalmology, Hadassah-Hebrew University Medical Center, Jerusalem 91120, Israel; 2 Department of Biochemistry and Molecular Biology, Institute of Medical Research Israel-Canada (IMRIC), The Hebrew University-Hadassah Medical School, Jerusalem 91120, Israel; 3 Department of Biotechnology, Hadassah Academic College, Jerusalem 9101001, Israel

**Keywords:** uveal melanoma, CREB, HIF-1, replication competent retroviral (RCR) vectors, therapy, metastasis

## Abstract

Uveal melanoma (UM) is the most prevalent primary intraocular cancer in adults. Up to half the patients develop metastases that are currently incurable, and most patients die within two years following the diagnosis of metastases. Therefore, novel therapeutic approaches are required. It has been established that tumor cells are more resistant to the hypoxia cue than non-malignant cells and can remain viable in hypoxia. Oxygen absence in hypoxic tumor areas means the absence of chemotherapeutics and the absence of the effector for radiotherapy (free oxygen radicals). To overcome this treatment resistance, we constructed MuLV-based replication-competent retroviral (RCR) vectors expressing shRNA targeting the hypoxia-response regulating genes *CREB* and *HIF-1*. These RCRs express shRNAs either against a single exon or against an exon and the poly-A signal to minimize the point-mutation resistance. These RCRs that only infect replicating cells will preferentially infect tumor cells. Pre-infected Mel270 UM subcutaneous xenografts in SCID mice were monitored weekly in vivo via bioluminescence. Here, we demonstrate that the knockdown of *CREB* or *HIF-1* in UM cells dramatically decreases UM tumor progression. The reduction of the expression of *Glut-1*, which is a major glucose transporter in cancer cells, within tumors that are infected with the armed viruses may indicate UM’s dependence on glycolysis for tumor progression.

## 1. Introduction

Uveal melanoma (UM) constitutes about 5% of all melanomas (based on Aronow et al. [1] and current Surveillance, Epidemiology, and End Results Program (SEER) data (https://seer.cancer.gov/)), and it is the most prevalent primary intraocular cancer in adults, with an annual incidence of 4–7 per million [1,2]. Overall, metastases appear in up to 50% of patients [3]. There is currently no effective treatment for the metastases, and most patients die within two years (80% in the first year and 92% in the second) [4]. Both primary and metastatic UM are relatively resistant to systemic chemotherapy and radiotherapy with only a modest efficacy [5,6]. Therefore, novel therapeutic approaches are required. 

A variety of studies established that tumor cells are more resistant to hypoxia than non-malignant cells and they can remain viable in hypoxic environments [7]. Moreover, experimental and clinical data have shown that tumor hypoxia is associated with tumor progression to a more malignant phenotype and with the spread of metastases, altogether resulting in poor survival in patients with various solid tumors [7,8]. Additionally, tumor hypoxia has been considered as a potential therapeutic problem, both because there is insufficient diffusion of chemotherapy in the hypoxic region and because radiation will not produce its effector—the free oxygen radical—without oxygen [9]. On the other hand, the on-going development of hypoxic regions in growing tumors provides an opportunity for tumor-selective therapies. 

To overcome the problem of these hypoxic treatment-resistant regions, we constructed MuLV-based replication competent retroviral (RCR) vectors [10] expressing shRNA targeting the hypoxia-response regulating genes. 

The adaptive response to hypoxia is orchestrated by a family of transcription factors (TFs), such as the hypoxia-inducible factors-1 (HIF-1) and -2 (HIF-2) [11], cyclic AMP-response-element (CRE) binding protein (CREB) [8], and the activator protein-1 (AP-1) [9].

HIF-1 is a heterodimer that is composed of an oxygen-regulated 1α subunit and a constitutively expressed 1β subunit. Non-hydroxylated active HIF-1 regulates the expression of hundreds of genes that are implicated in events, such as angiogenesis, cell survival/death, metabolism, migration, and metastasis. The genes that are involved in these processes are targeted by HIF-1, which binds to the hypoxia response elements (HRE). In the presence of oxygen, HIF-1 and HIF-2 are subjected to hydroxylation by prolyl-4-hydroxylase domain proteins. Under hypoxic conditions, the rate of hydroxylation declines such that non-hydroxylated proteins accumulate (for review, see Majmundar et al. [11]).

The HIF-1α and -2α subunits differ in their transactivation domains, which implies that they have unique target genes, although HIF-1α and -2α are highly conserved at the protein level, have similar domain structure, dimerize with HIF-1β, and activate HRE-dependent gene expression [11]. Indeed, it was shown that HIF-1 and HIF-2 have distinct, tissue specific expression patterns. HIF-1α is ubiquitously expressed, whereas HIF-2α expression is limited to the endothelium, kidneys, lungs, heart, and small intestine. HIF-1α has been shown to have a role in UM tumor progression and metastases formation [12,13]. 

Various mediators activate CREB through the phosphorylation of serine 133 (Ser133), which leads to the activation of CRE mediated transcription. CREB overexpression relative to adjacent normal tissues was found in many solid tumor types, such as non-small lung carcinoma (NSCLC), glioblastoma, mammary carcinoma, hepatocellular carcinoma (HCC), and melanoma [8,14,15]. This could be influenced by the modulation of signaling cascades upstream of CREB as well as the induction of CRE-dependent gene expression downstream of CREB, which leads to increased tumor growth, prevention of cell death, and enhanced metastasis formation and angiogenesis [8,12,13]. Furthermore, the overexpression of CREB in tumors often correlates with disease progression, poor patient survival, and chemotherapy resistance. We have previously demonstrated that the overexpression of a dominant-positive, hypoxia-insensitive CREB300/310 mutant enhanced tumor growth, vascularization, and abrogated hypoxia-induced apoptosis, while the dominant-negative form of CREB (KCREB) or knock-down of CREB abolished mouse hepatocellular carcinoma (HCC) tumor growth [8,14]. 

Similarly, we found that the infection of UM cells (both primary and metastatic cell lines) with a MuLV-based RCR that expresses shRNA targeting CREB (vACE-CREB) [14] reduces their survival in normoxic conditions by 50% [16]. The UM cells were found to have an increased apoptotic fraction in hypoxia that did not involve Caspase 3 activation and did not result in increased cell mortality. CREB knockdown increased UM cells’ sensitivity to chemotherapy (decarbazin—DTIC and doxorubicin—DOX), as in the HCC cells, an effect that was stronger in hypoxia [16]. 

The advantage of using this vector is that MuLV retroviruses only infect replicating cells, such as tumor cells, which generates a stably integrated provirus in the cells. These cells produce viral particles that express shRNA targeting CREB or HIF-1 that will spread within the tumor [14]. A point mutation in the target sequence against which we design shRNAs can result in tumor resistance to the treatment. To diminish resistance to the siRNA, we constructed RCR vectors expressing shRNA targeting two sites in each gene. The poly(A) signal is a conserved site in genes and less likely to be mutated. We have previously found that the poly(A) signal and the flanking nucleotides (−10 AAUAAA +10) are unique for each gene in the human genome (unpublished data, http://bioinfo.ekmd.huji.ac.il/honigman). Targeting siRNA to this region results in a very efficient knockdown. Based on these results, we constructed MuLV vectors expressing shRNA targeting the poly(A) signal, in addition to knocking down CREB via a sequence from exon 8 or targeting the poly(A) signal of HIF-1 in addition to targeting the exon 5 sequence. 

In the present work, we aimed to test whether knockdown of either CREB or HIF-1 in UM mouse xenograft models will be as effective at attenuating UM tumor growth in vivo as is the knockdown of these genes at reducing UM cell survival in vitro and in HCC growth in vitro and in vivo. In this work, we demonstrated that both regulators of the cellular response to hypoxia play a pivotal role in UM tumor progression and that CREB may play a more important role than HIF-1 in UM tumor progression.

## 2. Results

### 2.1. Construction of the RCR Vectors

**Figure 1:**
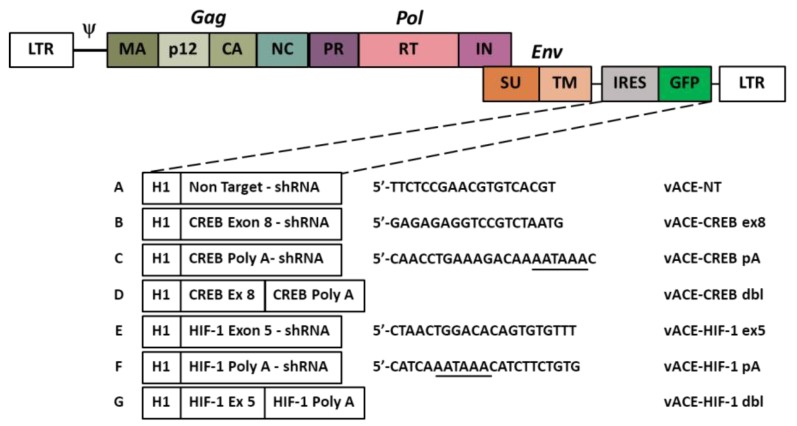
Schematic presentation of the various shRNAs recombinant replication competent retroviruses (RCRs). The IRES-*GFP* cassette from vACE was replaced by a H1 promoter which drives the expression of shRNAs targeting CREB or HIF-1. (**A**) A non-target (NT) sequence was used as control sequence for the effect of vACE alone on the tumors. (**B**,**E**) shRNA sequences targeting CREB (**B**) and HIF-1 (**E**) [14,16]. (**C**,**D**,**F**,**G**) To increase knockdown efficiency and minimize resistant mutations, we added shRNAs against the poly(A) signal (underlined) of each target. (**D**,**G**) Armed retroviruses carrying two shRNA sequences against the same target were termed: “double” (dbl).

We used the Murine Leukemia virus (MuLV). Viral construction and spread has been previously described [14,16]. In short, we replaced the IRES-GFP DNA fragment in vACE-GFP [10] by an H1 promoter that drives shRNA sequences to target CREB (vACE-CREB), HIF-1 (vACE-HIF-1), or a non-target sequence (vACE-NT). To reduce the chance of appearance of resistance to the knockdown, we constructed viruses armed with two shRNA sequences that target not only a unique exon sequence, but also the poly-A signal of each gene, termed double (dbl). See Figure 1 for the sequences of the shRNAs. 

### 2.2. Kinetics of Infection of Mel270 and MP41 by the RCR Vectors

**Figure 2:**
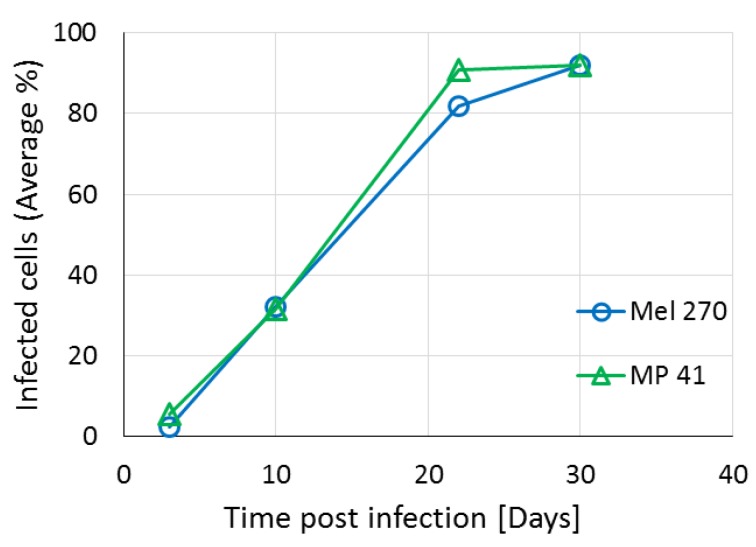
vACE-GFP spread in UM cell lines. The UM cell lines Mel270 and MP41 were infected by vACE-GFP. The cells were harvested every 6–7 days and the percent of infected cells was determined by flow cytometry. Readings were processed by the FCS Express 4 Flow Research Edition. The standard deviations are smaller than the graph’s resolution.

To determine the efficiency of infection of the two UM cell lines by the MuLV-based RCRs, we infected the cells with vACE-GFP. The spread of the virus was determined by flow cytometry (Figure 2). The kinetics of spread of the virus vector was similar in both of the cell lines and within 30 days post infection about 90% of the cells were infected. Based on these results, fully infected cell lines with the various armed viruses (Figure 1) were prepared based on these results. 

### 2.3. Knockdown Efficiency of the Double Targeting Vectors

We have previously demonstrated that RCRs targeting a single site in either CREB or HIF-1 efficiently reduce the level of the mRNA and the proteins of these genes in HCC and UM cell lines. Knockdown of the mRNA in the various cell lines was between 65% and 97% and protein levels of the targeted genes were reduced by 56% to 86% [14,16].

**Figure 3:**
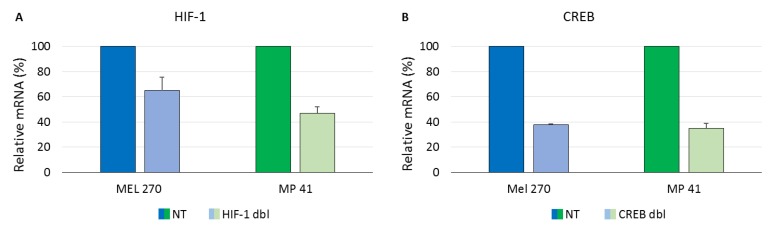
Quantification of knockdown of HIF-1 (**A**) and CREB (**B**) by the armed viruses. mRNA was extracted from fully infected cell lines and mRNA of the two genes was quantified by RT-qPCR relative to β-actin and relative to the expression of these genes in the vACE-NT infected cells, as previously described [16].

To evaluate the efficiency of knockdown of the double targeting recombinant viral vectors, we determined the reduction in mRNA levels, the target of the shRNAs. The efficiency of knockdown of HIF-1 and CREB by RCRs targeting two sites in either gene was determined by RT-qPCR mRNA analysis, as previously described for the targeting with only one sequence of each of these genes [16]. The double-targeting armed viruses efficiently knocked down both CREB and HIF-1 (about 50–60%) in both cell lines (Figure 3), similar to the knockdown that was achieved by targeting a single site in the genes [16]. Keeping in mind the advantage of reduction in resistance to shRNA by targeting two sites in a single gene, we continued our experiments with the double targeting viral vectors.

### 2.4. Kinetics of Tumor Growth

**Figure 4:**
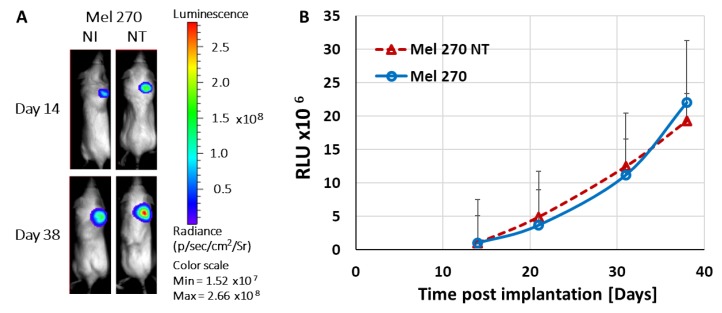
Kinetics of tumor growth. Not infected (NI) Mel270 and Mel270-stably infected with vACE-NT (Mel270 NT) stably expressing the *luc* gene were injected subcutaneously in SCID mice (*n* = 5 in each group). Growth of tumors was monitored over 38 days via bioluminescence while using the IVIS camera, as previously described [14]. (**A**) Pictures of one representative mouse per group; (**B**) Tumor growth rate expressed by relative light units (RLU).

For the in vivo mouse xenograft model experiments, we utilized cells that stably express the luciferase gene (*luc*) that enables in vivo non-invasive monitoring of tumor growth along the time span of the experiment. To determine if the MuLV vector by itself affects tumor growth we compared the growth rate of non-infected cells with that of cells stably-infected with the control RCR (vACE-NT) vector. Infection with vACE-NT did not affect tumor growth (Figure 4).

**Figure 5:**
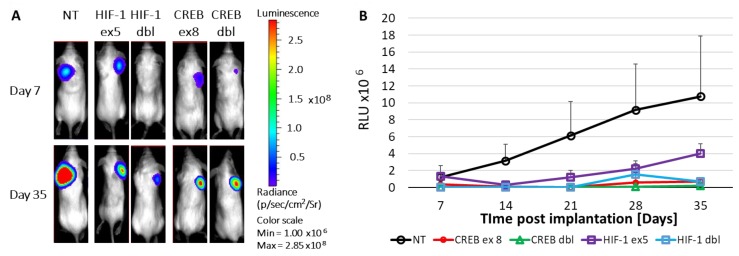
The role of CREB and HIF-1 in tumor progression. Mice (*n* = 5 in each group) were injected subcutaneously with 4 × 10^6^ Mel270 UM cells stably expressing the *luc* gene and either of the shRNAs targeting CREB or HIF-1 via a single or a double shRNA expressing RCRs. Cells expressing the non-target (NT) shRNA sequence served as control. Tumor growth was monitored via bioluminescence as previously described [16]. (**A**) A representative mouse from each group is depicted at day 7 and day 35 post transplantation. (**B**) Kinetics of growth of the UM tumors infected by the various armed viruses expressed by relative light units (RLU). The growth rate of tumors infected with vACE NT is significantly greater than that each of all the other armed viruses (*p* < 0.0001 for each comparison and for the entire model).

Tumors infected with the armed viruses that knock either CREB or HIF-1 show a flat growth curve as opposed to the steady fast growth of the control tumors (Figure 5). These results indicate that both regulators of the cellular response to hypoxia play a pivotal role in UM tumor progression.

**Figure 6:**
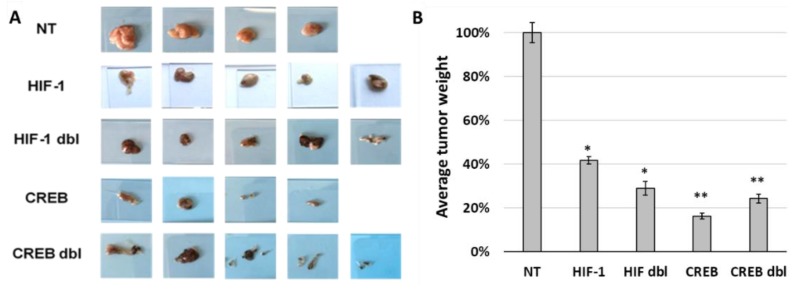
Effect of knockdown of either CREB or HIF-1 on tumor growth. Uveal melanoma tumors excised on day 35 post implantation (Figure 5) were photographed (**A**). The tumors were weighed and the average weight of each group is presented relative to the average weight of UM tumors infected with vACE-NT (**B**). Comparing each group to NT (*t* test) demonstrated a statistically significant decrease in tumor size for all the groups (* *p* < 0.5; ** *p*< 0.01).

At the end of the experiment, the tumors were excised, photographed and weighed (Figure 6). Tumors infected with the armed viruses were smaller than the tumors infected with the control RCR (vACE-NT, Figure 6 left panel). The mean weight of the tumors infected with an armed virus knocked down HIF-1 was only 42% of the mean tumor weight of the control tumors, and the mean weight of the tumors infected with an armed virus that knocked down CREB was only 16% of the mean control tumor. These results support those of the in vivo monitoring of tumor growth (Figure 5) and suggest that CREB may play a more important role than HIF-1 in UM tumor progression.

### 2.5. Histopathological Analysis of the Tumors

**Figure 7:**
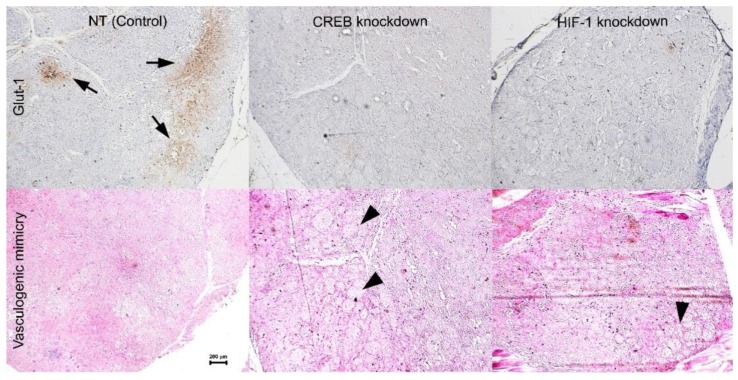
Histopathological analysis. Uveal melanoma tumors excised on day 35 (Figure 5) were processed for routine histopathology and serial sections were stained with H & E, anti-CD34, anti-Glut-1, and with PAS without hematoxylin (to highlight vasculogenic mimicry). Analysis of histopathological stains on five tumors per group (see Figure 6) was carried out by three independent viewers (magnification ×4, scale bar 200 microns).

Histopathological analysis of the excised tumors inspected by three independent reviewers (S.F., M.G.V. and S.L.) revealed solid tumor masses with hardly any necrosis in any of the tumors. Sections were reviewed and graded for the absence (0), presence (1) or abundance (2) of staining for blood vessels (anti-CD34), vasculogenic mimicry patterns [17] and Glut-1 expression. Immunohistochemistry (IHC) for the endothelial cell marker CD34 did not highlight a vascular network within any of the tumors, but staining for vasculogenic mimicry with PAS without hematoxylin highlighted back-to-back loop (networks) mostly in tumors infected with the armed viruses targeting CREB, while most of the tumors infected with the control vACE-NT vector did not show this cellular pattern. Glut-1 is expressed by UM cells in hypoxia [16] and its expression is regulated by CREB [18] and HIF-1 [19]. Indeed, NT tumors showed patches of areas expressing Glut-1 that did not appear in any of the tumors with knockdown of either CREB of HIF-1 (Figure 7).

## 3. Discussion

Ongoing generation of hypoxic regions is a property of solid tumors. The cellular response to hypoxia is orchestrated by several transcription factors including the HIF family and CREB. In a previous work, we demonstrated in vitro that knockdown of CREB in hypoxia, following infection with vACE-CREB, increased apoptosis by 50% and decreased the expression of CREB-regulated genes such VEGF in two UM cell lines Mel270 and OMM2.5 [16]. In this work we demonstrate for the first-time inhibition of uveal melanoma growth in a mouse model, following knockdown of either CREB or HIF-1 by a replication competent retrovirus vector. This vector can integrate stably and spread within the infected tumor.

To diminish resistance to the knockdown by shRNA expressed by the armed MuLV vectors we added to each vector another shRNA targeting the poly(A) site of each gene. Thus, each vector expressed two shRNAs from the same RNA molecule targeting two sites in each gene. As demonstrated in Figure 3 and Figure 5, these recombinant vectors efficiently knocked down both CREB and HIF1 in vitro and abolished tumor growth in vivo. There is a debate in the literature regarding the relevance of long-standing cell lines to the primary tumors from patients. To address this concern, we compared in vitro virus infectivity and spread and knockdown of the target genes in three different UM cell lines: Mel270, OMM2.5 and the recently prepared MP41 cell line which has been shown to have the same gene expression profile as the tumor from which it originated from [20]. In the in vitro experiments, we did not find significant differences in the response of the cells to the infection with the armed viruses.

Tumor progression of subcutaneously implanted xenografts stably expressing shRNA targeting either CREB or HIF-1 was diminished as determined by both non-invasive luciferase-based imaging and measurements of the excised tumors (Figure 5 and Figure 6). These results suggest that even partial knockdown (about 50% in vitro) of either one of these transcription factors had a major effect on tumor progression. Moreover, we have previously shown in hepatocellular carcinoma that knockdown of either CREB or HIF-1 reduced the expression of VEGF within the tumors which resulted in a decrease in hypoxia-guided neovascularization [14]. The abrogation of VEGF may be responsible for the stronger effect in UM seen in vivo in the current study compared to the in vitro results in our previous study [16].

UM is well known for its poor internal vasculature, which led to the discovery of vasculogenic mimicry [21]. We now know that tumor cells transform into endothelial-like cells and form channels that enable plasma and nutrients to flow throughout the tumors [17]. The histopathologic analysis in the current study indicates that formation of vasculogenic mimicry may be inversely correlated with the activity of CREB (Figure 7).

Tumors infected with the control vector, grew rapidly with the expected formation of hypoxic areas within the tumors where Glut-1 was expressed (Figure 7). However, when the expression of either CREB or HIF-1 was knocked down, the expression of their downstream gene *glut-1* was abolished. Glut-1 is the major glucose transporter, especially in tumor cells and is important in providing glucose for energy production within these cells [22]. Activation of Hexokinase II is the initial and rate limiting step in glycolysis. Liu and colleagues found that Hexokinase II is activated by CREB in UM cells. In addition, downregulation of Hexokinase II resulted in reduced glycolysis, increased oxygen consumption and reduced tumor growth [23]. In vitro, UM cells have sufficient supply of glucose in the growth medium, even in hypoxic conditions, which enables them to survive hypoxia. However, in vivo, glucose is not readily available for the tumor cells. Following the knockdown of either CREB or HIF-1, there was a reduction in Glut-1 expression resulting in limited energy supply preventing UM tumors proliferation in hypoxia. This could be at least part of the reason these tumors hardly grew in vivo (Figure 5, Figure 6 and Figure 7). Taken together with our previous results [16], it appears that the role played by CREB and HIF-1 in UM is more important in vivo than in vitro.

In cutaneous melanoma there have been publications linking the level of pigmentation to the virulence of the tumor and to HIF-1 activation [24,25]. Uveal melanoma is vastly different to cutaneous melanoma in both genetic profile and clinical behavior. Uveal melanoma cell lines do not produce pigment in vitro precluding a proper analysis of the role of melanin. There are no studies correlation the level of pigmentation and HIF-1 in UM cells and their virulence. One study correlated pigmentation with survival of UM patient [26]. In that study, although there was no difference in prognosis between races, and despite a similar distribution of pigmented vs. non-pigmented tumors clinically, the authors found that in Caucasians more pigmented tumors correlated with a higher metastatic rate. Of note, it is well known that when eyes with uveal melanomas are enucleated, a pigmented surface that would give a higher pigmentation score clinically, can sometimes cover an internal amelanotic tumor. In this work we demonstrate that HIF-1 plays a role in UM tumor progression in vivo.

Our previous results [16], demonstrated that knockdown of CREB resulted in increased sensitivity to chemotherapeutic agents (doxorubicin and decarbazin). This, together with our findings in the present study, suggest that a combined treatment with the armed viruses and chemotherapy should result in a more potent effect in the reduction of tumor progression.

## 4. Materials and Methods

### 4.1. Cell Culture

The human Mel270 cell line [27] (verified by STR analysis and a kind gift from Prof. Sarah Coupland, Liverpool, UK) and MP-41 [20] were grown in RPMI 1640 supplemented with 10% fetal bovine serum, 2 mM glutamine, 100 IU/mL penicillin, 100 μg/mL streptomycin (Biological Industries, Kibbutz Beit-Haemek, 25115, Israel) and incubated at 37 °C in a humidified atmosphere with 5% CO_2_.

### 4.2. Plasmids and Viruses

The plasmid pACE-GFP (a kind gift from Prof. Noriyuki Kasahara, Los Angeles, California [10]) contains a full-length replication-competent amphotropic MuLV provirus with an additional internal ribosome entry site (IRES)-*GFP* cassette flanked by BsiWI and NotI restriction enzymes sites. This cassette was replaced by oligonucleotides harboring the H1 promoter driving the transcription of the following shRNA sequences:

5′_GAGAGAGGTCCGTCTAATGTTCAAGAGACATTAGACGGACCTCTCTCTTTTT **(pACE-CREB)**

5′_GAGAGAGGTCCGTCTAATGTTCAAGAGACATTAGACGGACCTCTCTCTTTTTCAACCTGAAAGACAAAATAAAC (**pACE-CREB dbl)**

5′_CTAACTGGACACAGTGTGTTTAATATATGAAAACACACTGTGTCCAGTTAGTTTTTT (**pACE-HIF-1**)

5′_CTAACTGGACACAGTGTGTTTAATATATGAAAACACACTGTGTCCAGTTAGTTTTTTCATCAAATAAACATCTTCTGTG (**pACE-HIF-1 dbl**)

5′_ACCAAGATGAAGAGCACCAACCTGAACCATTGGTGCTCTTCATCTTGGTTTTTTT (**pACE-NT, non-target shRNA**)

See Figure 1 for a schematic presentation of the vectors.

### 4.3. Virus Preparation

HEK293T cells were transiently transfected with either one of the pACE plasmids, described above and in Figure 1, using FuGENE HD reagent (Promega-Corp, Madison, WI, USA). The medium containing the virus particles was harvested 48 h later, filtered (MILLEX-HV, PVDF 0.45 µ) and stored at −80 °C.

### 4.4. Quantitative Real-Time PCR

RNA was extracted from the cells using the NucleoSpin^®^ RNA (Macherey-Nagel, Bethlehem, PA, USA), according to the manufacturer’s instructions. The purified RNA samples were subjected to reverse transcription using GoScript (Promega-Corp, Madison, WI, USA), monitored by quantitative 7900HT real-time PCR apparatus (Applied Biosystems, Foster City, CA, United States) utilizing the GoTaq Real-Time PCR reagents (Promega-Corp, Madison, WI, USA) and the following specific primers:

**CREB:**

fp-5′_CCCAGCACTTCCTACACAGCCTGC_3′,

rp-5′_ CGAGCTGCTTCCCTGTTCTTCATTAGACG _3′.

**HIF-1:**

fp-5′_GGGATTAACTCAGTTTGAACTAACTGG_3′,

rp-5′_CCTTTTTCACAAGGCCATTTCTGTGTG_3′.

The results were normalized to the cellular house-keeping gene.

**β-actin:**

fp-5′_CCTTCCTGGGCATGGAGTCC_3′,

rp-5′_GTGTTGGCGTACAGGTCTTTGC_3′.

### 4.5. Flow Cytometry (FACS)

Cells infected with vACE-GFP were harvested every seven days, centrifuged (4 °C) at 1750 rpm for 4 min. The supernatant was removed and the cells were re-suspended in PBS +1% FCS, then filtered through a Mesh filter and run on the FACS machine (BD FACScan analyzer, 3A International Business Park, Singapore). Results were analyzed using FCS Express 4 Flow Research Edition (De Novo Software, Glendale, CA, USA, Version 4.07.0020).

### 4.6. Xenograft Mouse Model

Mel270 cells stably expressing *luc* and infected with one of the RCR recombinant viruses (Figure 1) were harvested and 4 × 10^6^ cells in 100 μL were injected subcutaneously (SC) above the foreleg of severe combined immunodeficiency (SCID) mice (C.B-17/ICRHSD-PRKDC-SCID) about three weeks of age (weighing 20–24 gr). To allow descriptive statistics, each group included five mice. Male and female mice were randomly assigned to each treatment group. Each group included a similar distribution of sexes. Tumor growth was monitored weekly following IP injection of D-Luciferin 300 mg/0.1 cc (PBS)/mouse (Promega-Corp, Madison, WI, USA) 10 min. before imaging. This non-invasive luciferase-based method for monitoring tumor growth allows following the same tumor in the same mouse over time and minimizes the number of mice used in the experiment. Mice were anesthetized with isoflurane and bioluminescence was measured with the IVIS In Vivo System (Caliper Life Sciences, Wlatham, MA, USA). No animals were excluded from the analysis, and there was no blinding during the experiments. The IACUC of the Hebrew University of Jerusalem approved the animal protocol used in this study (NIH approval number OPRR-A01-5011 from 29 June 2016).

### 4.7. Immunohistochemistry

At the end of the experiments, the tumors were excised, photographed, measured, and fixed in 4% formaldehyde for routine processing and embedding, as previously described [14]. Four-micron thick sections were cut and stained with hematoxylin–eosin (H & E) or with periodic acid Schiff (PAS) without hematoxylin. Paraffin sections were cut at 4 μm for light microscopic immunohistochemistry (IHC). The slides were deparaffinized using xylene and absolute ethanol, rinsed in distilled water, and exposed to H_2_O_2_ for 5 min. or antigen unmasking solution. The antigen unmasking solution (citrate buffer, Thermo Scientific, Waltham, MA, USA) was heated in a steamer to 105 °C for 10 min. and then cooled to room temperature before use. The sections were rinsed with PBS for 2 min. (Cell Marque, Rocklin, CA, USA) and then blocked with CAS-Block for 5 min. (Invitrogen, Carlsbad, CA, USA, by ThermoFisher Scientific, Waltham, MA, USA). The slides were rinsed with PBS and reacted with primary antibodies targeting CD34 (Cell Marque), and Glut-1 (Cell Marque), followed by rinsing in PBS and reaction with secondary antibodies: MACH-2 rabbit HRP-Polymer (Biocare Medical, Pike Lane Concord, CA, USA) and HRP linked to rabbit anti-FITC, respectively. Sections were rinsed with PBS and incubated with AEC (Cell Marque) for 10 min. Sections were rinsed in distilled water and PBS and counterstained with Mayer’s hematoxylin, and coverslipped with a permanent mounting medium (AquaSlip, American MasterTech, Lodi, CA, USA). The slides were photographed with a Nikon ECLIPSE Ti microscope at ×4 magnification.

### 4.8. Statistical Analysis

*In vitro* experiments with at least three biological and three technical repeats for each data point provide sufficient statistical power. For animal experiments, we used five animals per group to allow descriptive statistics on the one hand while minimizing the use of animals to accommodate the ethical restrictions on the other hand.

Statistical analysis was performed with JMP 9.0 (SAS, Cary, NC, USA). Analysis of variance (ANOVA) was used to compare the mRNA levels and luciferase activity in relative light units. Multivariate ANOVA was used to compare the growth rates of the tumors within the mice (Figure 5 and Figure 6).

## 5. Conclusions

Clinical and research studies have shown that uveal melanoma is resistant to hypoxia. We have confirmed UM’s resistance to hypoxia in vitro [17], and used armed viruses that knock down the expression of the regulators of cellular response to hypoxia, CREB, and HIF-1 to learn whether inhibiting the cells’ response to hypoxia would result in diminished tumor growth. Despite the lack of response in vitro, here we found that knockdown of CREB or HIF-1 in vivo resulted in a markedly decreased tumor growth rate. The resulting tumors were smaller in size and weight, had no vasculature growing into them, and had increased expression of vasculogenic mimicry patterns (more so with the knockdown of CREB).

The expression of the major cancer glucose transporter Glut-1, which is regulated by CREB and HIF-1, was diminished in UM tumors following the knockdown of either CREB or HIF-1. The resultant reduction in glucose uptake together with lower availability of glucose in vivo vs. in vitro probably lead to the curbed growth of the UM cells in vivo. In summary, the results presented here, demonstrating that the knockdown of either CREB or HIF-1 reduced UM tumor growth, may lead to the development of a novel therapeutic approach for metastatic UM, which is currently incurable.
